# Oral Sub-Chronic Ochratoxin a Exposure Induces Gut Microbiota Alterations in Mice

**DOI:** 10.3390/toxins13020106

**Published:** 2021-02-01

**Authors:** María Izco, Ariane Vettorazzi, Maria de Toro, Yolanda Sáenz, Lydia Alvarez-Erviti

**Affiliations:** 1 Laboratory of Molecular Neurobiology, Center for Biomedical Research of La Rioja (CIBIR), 26006 Logroño, Spain; mizco@riojasalud.es; 2 MITOX Group, Department of Pharmacology and Toxicology, Faculty of Pharmacy, Universidad de Navarra, 31008 Pamplona, Spain; avettora@unav.es; 3 IdiSNA, Navarra Institute for Health Research, 31008 Pamplona, Spain; 4 Center for Biomedical Research of La Rioja (CIBIR), Genomics and Bioinformatics Core Facility, 26006 Logroño, Spain; mthernando@riojasalud.es; 5 Molecular Microbiology Area, Center for Biomedical Research of La Rioja (CIBIR), 26006 Logroño, Spain; ysaenz@riojasalud.es

**Keywords:** mycotoxin, ochratoxin A, gut microbiota, toxicity, mice

## Abstract

Gut microbiota plays crucial roles in maintaining host health. External factors, such as diet, medicines, and environmental toxins, influence the composition of gut microbiota. Ochratoxin A (OTA) is one of the most prevalent and relevant mycotoxins and is a highly abundant food and animal feed contaminant. In the present study, we aimed to investigate OTA gut microbiome toxicity in mice sub-chronically exposed to low doses of OTA (0.21, 0.5, and 1.5 mg/kg body weight) by daily oral gavage for 28 days. Fecal microbiota from control and OTA-treated mice was analyzed using 16S ribosomal RNA (rRNA) gene sequencing followed by metagenomics. OTA exposure caused marked changes in gut microbial community structure, including the decrease in the diversity of fecal microbiota and the relative abundance of *Firmicutes*, as well as the increase in the relative abundance of *Bacteroidetes* at the phylum level. At the family level, six bacterial families (unclassified *Bacteroidales, Porphyromonadaceae,* unclassified *Cyanobacteria, Streptococcaceae, Enterobacteriaceae, Ruminococcaceae*) were significantly altered by OTA exposure. Interestingly, OTA-induced changes were observed in the lower-dose OTA groups, while high-dose OTA group microbiota was similar to control group. Our results demonstrated that sub-chronic exposure at low doses of OTA alters the structure and diversity of the gut microbial community.

## 1. Introduction

Gut microbiota plays crucial roles in maintaining host health and regulating numerous physiological functions, not only in the gastrointestinal tract, but also in other organs as well as in the systemic immune system [1,2]. Given the close symbiotic gut microbiota–host relationship, dysbiosis of gut microbiota has been involved in the pathogenesis of multiple diseases [3,4]. Numerous animal and human studies have shown that dysbiosis of gut microbiota is associated with diseases as autism, asthma, cardiovascular disease, colon cancer, Crohn’s disease, diabetes, eczema, food allergies, irritable bowel syndrome, obesity, hepatic encephalopathy, and mental disorders [5,6,7]. Therefore, maintaining or regulating the balance of intestinal bacteria is essential for animal and human health, although several factors can influence the composition of gut microbiota. External factors, such as diet, medicines, and environmental toxins, influence the composition of the gut microbiota.

Mycotoxins are secondary metabolites produced by fungi and are capable of causing toxic, carcinogenic, and/or teratogenic effects in animals and humans [8,9]. Mycotoxins are among the most frequently occurring natural food contaminants in human and animal diets, leading to acute and chronic exposures in humans and animals [10,11]. The gastrointestinal tract is the first physiological barrier against mycotoxins as well as the first target for these toxics following ingestion of contaminated food. Given the initial interaction of mycotoxins with the gut epithelium, this topic has gained pronounced interest in the last decade and there is an increasing evidence of the adverse effects of various mycotoxins on vulnerable intestinal structures and intestinal integrity [12,13,14,15,16]. Moreover, on one hand, it is known that several bacteria have the ability to protect, mainly farm animals, from mycotoxin effects through metabolizing or binding to mycotoxins, but on the other hand, some studies have also shown the ability of mycotoxins to negatively affect the gut microbiota. However, the effect of mycotoxins on the gut microbiota has been poorly investigated [16,17].

Ochratoxin A (OTA) is one of the most prevalent and relevant mycotoxins produced by *Aspergillus* and *Penicillium* species [18]. OTA is a highly abundant food and animal feed contaminant detected in cereals, coffee, wine, beer, dried fruits and nuts, meat products, food coloring agents, and even in bottled water [19,20,21]. OTA has been shown to be carcinogenic [22], hepatotoxic [23], nephrotoxic [24], and immunotoxic [25]. Metabolization and accumulation of OTA mainly occurs in the liver and kidneys [26], with the kidneys being the main target organ for its toxicity in all mammalian species tested [27]. OTA has been reported to disrupt the intestinal absorption of nutrients, alter the intestinal cell function, and damage the intestinal cell integrity [14,28,29]. Data on OTA gut microbiome toxicity are limited and only a few studies have evaluated the effects of OTA on gut microbiota. Ouethrani et al. demonstrated that OTA significantly affects the metabolism of the colonic microbiota and reduces the relative abundance of beneficial microbes, such as *Lactobacillus* spp. and *Bifidobacteria* spp., in a dynamic simulation model of the descending human colon [30]. In an in vivo study with OTA-treated rats, OTA modified the relative abundance of *Bacteroidaceae* and *Lactobacillaceae* in gut microbiota [31]. Recently, Wang et al. found that OTA decreased the richness and diversity of the microbiota and the relative abundance of *Firmicutes* and also increased the relative abundance of *Bacteroidetes* and *Bacteroides* in ducks [32]. These results were confirmed by Zhai et al., who further demonstrated that OTA also alters the metabolism of intestinal microbiota in ducks [33]. Changes in the diversity and in the composition of gut microbiota caused by OTA have also been found in OTA-fed broilers [34] and turkeys [35].

Although the mouse is the most commonly used organism in basic and experimental research, only one study has investigated the effect of OTA on the growth of colonic probiotic bacteria in mice [36]. However, culture-dependent methods have limitations since the majority of intestinal bacteria are widely considered to be unculturable and have never been isolated in the laboratory. Therefore, the present study aimed to investigate the impact of OTA on the gut microbiota in mice exposed for 28 days to OTA by daily oral gavage using 16S ribosomal RNA (rRNA) gene-based metagenomic analysis. We found that OTA exposure caused marked changes in the gut microbial community structure, including the decrease in the diversity of fecal microbiota and the relative abundance of *Firmicutes*, as well as the increase in the relative abundance of *Bacteroidetes* at the phylum level. At the family level, six bacterial families were significantly altered by OTA exposure.

## 2. Results

**Figure 1:**
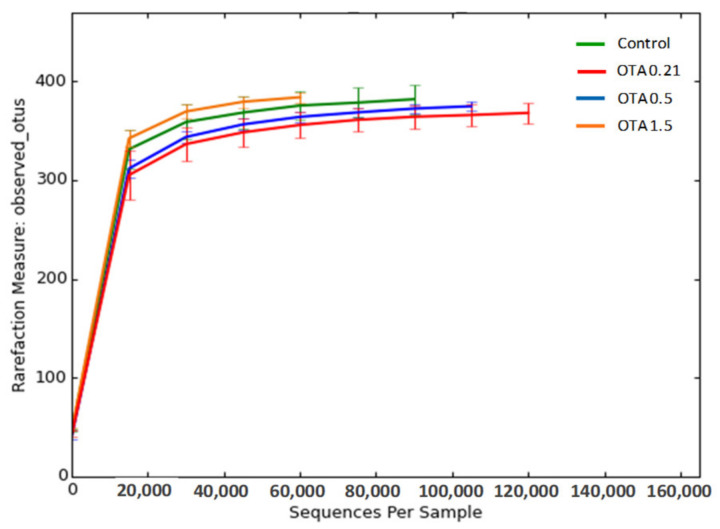
Rarefaction curves of observed operational taxonomic units (OTUs) for each group. The *x*-axis represents the number of sequences per sample and the *y*-axis refers to observed number of OTUs at 97% sequence similarity. The plateau indicates that the fecal microbial community was fully detected with the sequencing depth used. Green, red, blue, and orange lines indicate the control, OTA 0.21-treated group, OTA 0.5-treated group, and OTA 1.5-treated group (mg/kg body weight), respectively.

To investigate the effect of oral sub-chronic exposure to OTA on intestinal microbiota, we analyzed fecal microbiota from control and OTA-treated mice using 16S rRNA gene sequencing and subsequent metagenomics analysis. After removing unqualified sequences, a total of 2,543,861 high-quality reads were generated from a total of 15 fecal samples from the different groups (for control, OTA 0.5, and OTA 1.5, *n* = 4 samples; and for OTA 0.21, *n* = 3), with each fecal sample producing an average of 169,590 ± 15,035 (means ± standard deviation) effective sequences. On the basis of a 97% similarity level, we clustered all the effective reads into operational taxonomic units (OTUs). The rarefaction curves of the four groups reached a plateau, indicating that the sequencing depth used in this study was adequate in evaluating the microbial diversity of each sample and in precisely describing the fecal microbial communities (Figure 1).

**Figure 2:**
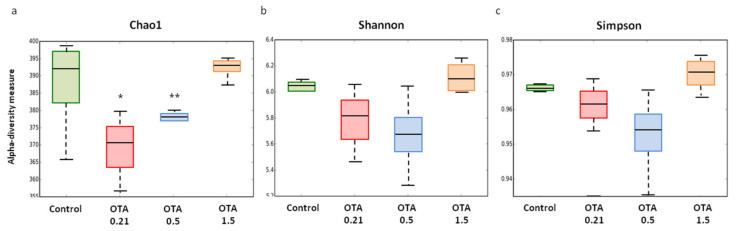
Box plots showing alpha diversity indexes (Chao1, Shannon, and Simpson) in fecal microbiota of control and ochratoxin A (OTA)-treated mice. Chao1 index (**a**) reflects the OTU richness in samples, while Shannon (**b**) and Simpson (**c**) indexes reflect the evenness and global diversity of OTUs, respectively. Values are expressed as median (*n* = 3–4). Differences were assessed by non-parametric Kruskal–Wallis test, * *p* <0.05; ** *p* <0.01 compared to OTA 1.5.

Alpha diversity reflects the species richness and diversity of a single sample, which is determined by several indexes. Chao1, Shannon, and Simpson indexes were calculated, as Figure 2 shows. We used the Chao1 index to estimate the fecal microbial richness, and this index was significantly decreased in the lower-dose OTA groups compared to the higher-dose OTA group (OTA 0.21: *p* = 0.0351; OTA 0.5: *p* = 0.0029; Figure 2a). Compared to the control group, we also observed a decreased Chao1 index in the OTA 0.21 and 0.5 groups, although it was not statistically significant. These results suggested that OTA at doses of 0.21 and 0.5 mg/kg significantly reduced the number of microorganisms in the fecal samples. The evenness (Shannon index) and global diversity (Simpson index) of the fecal microbiota showed a downward trend in mice exposed to the lower doses of OTA compared to control and OTA 1.5-treated mice, but the differences were not significant (*p* > 0.05, Figure 2b,c).

**Figure 3:**
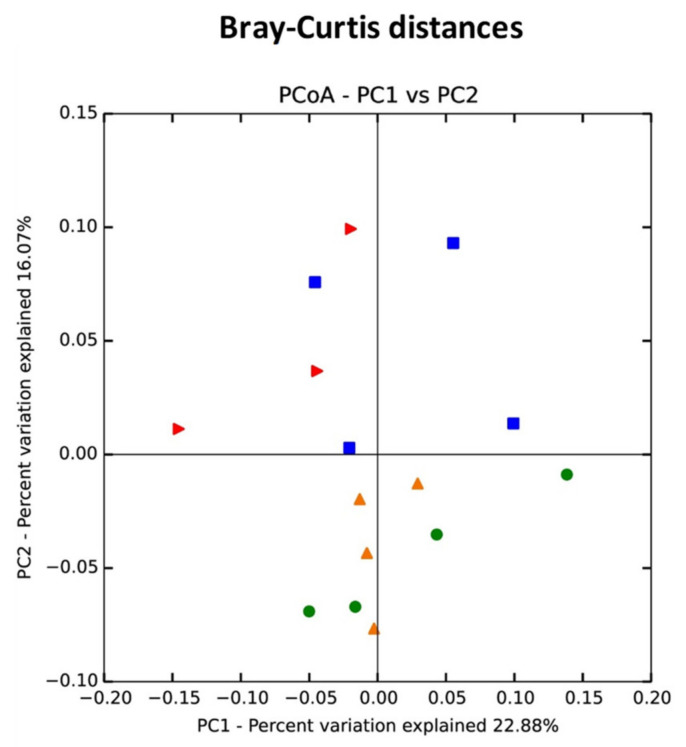
Principal coordinate analysis (PCoA) analysis of fecal microbiota from control and OTA-treated mice based on Bray–Curtis distances. A distinct clustering of samples by OTA treatment was observed. The percentage of variation explained by PC1 and PC2 are noted in the axes. Groups are distinguished by colors. Each colored symbol corresponds to an individual sample.

Beta-diversity shows the variations of microbial communities between samples. Regarding beta-diversity, principal coordinate analysis (PCoA) plots based on Bray–Curtis dissimilarity showed that fecal microbial communities from control and OTA 1.5-treated mice were more similar to each other than to those from lower-dose OTA mice (Figure 3). Furthermore, these groups were clearly separated into different clusters, as shown by PC2, indicating that oral exposure to OTA at doses of 0.21 and 0.5 mg/kg altered fecal microbiota community structure in mice.

**Figure 4:**
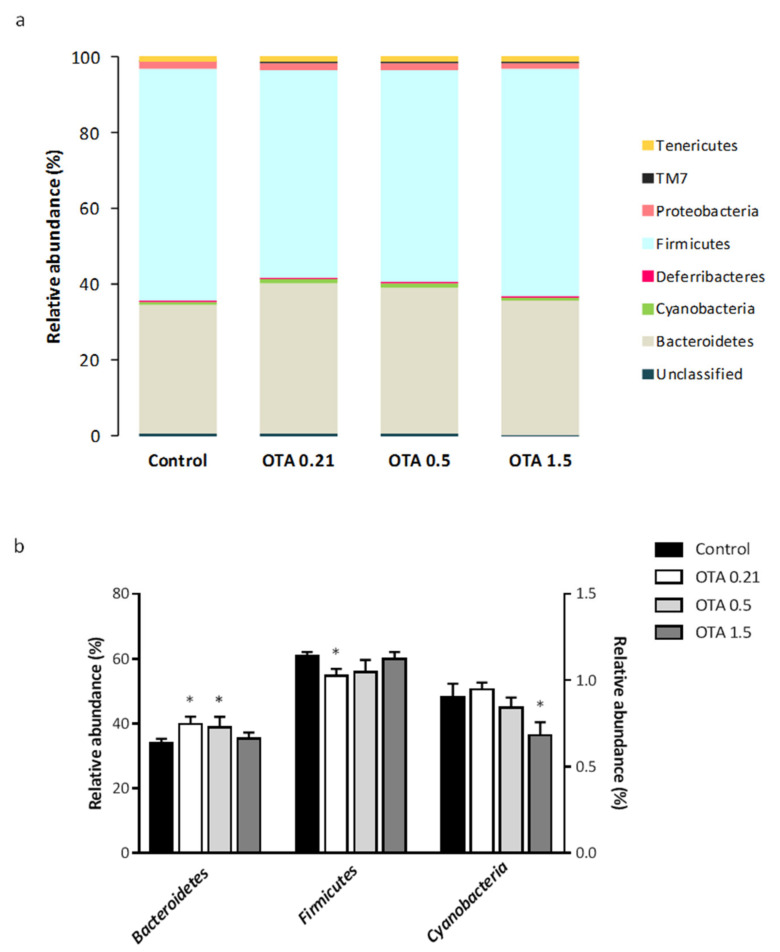
Differential analysis of microbial composition at the phylum level. (**a**) Column plots of relative abundance of the identified phyla. Different colors represent different phyla; the percentage on the vertical axis indicates the relative abundance of each bacterial phyla. (**b**) Fecal microbial abundances with significant differences between control and OTA-treated groups at the phylum level. Data from *Cyanobacteria* refers to the right *y*-axis. Data are shown as mean ± standard error of the mean (SEM) (*n* = 3–4). Differences were assessed by non-parametric Kruskal–Wallis test followed by Mann–Whitney *U* test; * *p* <0.05 compared to control mice.

To explain more detailed changes in the fecal microbial structure induced by OTA exposure, we determined the relative abundance at the phylum and family levels. At the phylum level, a total of eight bacterial phyla were identified in all fecal samples, with *Bacteroidetes* and *Firmicutes* being the most abundant phyla in the feces of mice from the four groups, accounting for about 38.7% and 57.9% of the bacterial abundance, respectively (Figure 4a). *Proteobacteria* and *Tenericutes*, which were represented by 1.8% and 1.3% of the total abundance, were the third and fourth highly abundant phylum, respectively. The remaining phyla constituted <0.8% of the bacterial abundance.

We found that OTA exposure differentially modified the fecal microbiota composition in mice, altering the abundance of three of the eight bacterial phyla identified. Specifically, the relative abundance of *Bacteroidetes* increased and the relative abundance of *Firmicutes* decreased significantly in the two lower-dose OTA groups compared to the control group (OTA 0.21: *p* = 0.034 for *Bacteroidetes* and *p* = 0.034 for *Firmicutes*; OTA 0.5: *p* = 0.043 for *Bacteroidetes* and *p* > 0.05, n.s. for *Firmicutes*; Figure 4b). The relative abundance of *Bacteroidetes* and *Firmicutes* in mice exposed to the highest dose of OTA remained similar to the relative abundance in control mice. *Cyanobacteria* showed a significant decrease in mice orally exposed to OTA at dose of 1.5 mg/kg compared to control mice, while no evident change occurred in the OTA 0.21 and OTA 0.5 mice. Thus, the relative abundance of *Cyanobacteria* accounted for 0.90% of the bacterial abundance in control mice, but was significantly down to 0.68% after OTA 1.5 treatment (*p* = 0.021 compared to control mice; Figure 4b).

**Figure 5:**
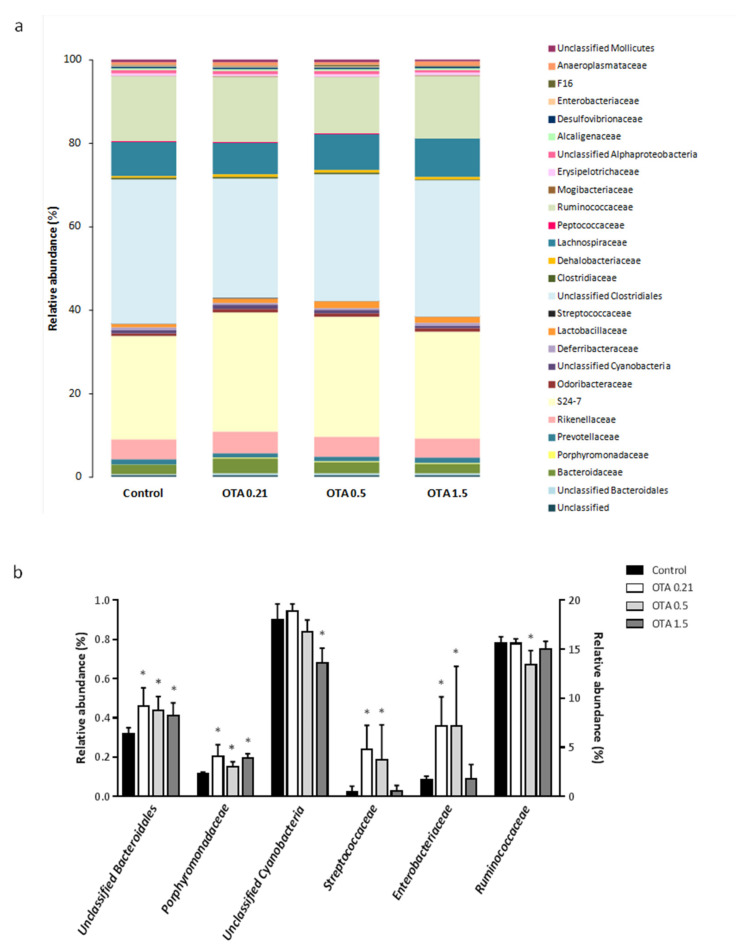
Differential analysis of microbial composition at the family level. (**a**) Column plots of relative abundance of the identified families. Different colors represent different families; the percentage on the vertical axis indicates the relative abundance of each bacterial family. (**b**) Fecal microbial abundances with significant differences between control and OTA-treated groups at the family level. Data from *Ruminococcaceae* refers to the right *y*-axis. Data are shown as mean ± SEM (*n* = 3–4). Differences were assessed by non-parametric Kruskal–Wallis test followed by Mann–Whitney *U* test; * *p* < 0.05 significantly different from control mice.

At the family level, a total of 28 families were detected. The relative abundances of the identified bacterial families are shown in Figure 5a and Table 1. Unclassified *Clostridiales, Muribaculaceae* (formerly “family S24-7”), *Ruminococcaceae*, and *Lachnospiraceae* were the dominant families detected in the four groups, accounting for about 31.6%, 26.9%, 14.9%, and 8.3% of the bacterial abundance, respectively. *Rikenellaceae*, *Bacteroidaceae*, and *Lactobacillaceae* comprised 4.8%, 2.7%, and 1.2% of the total abundance, respectively. The remaining families represented less than 1% of the bacterial abundance (Figure 5a and Table 1).

We found that six bacterial families were significantly altered by OTA exposure. Specifically, unclassified *Bacteroidales* and *Porphyromonadaceae* increased significantly in mice exposed to OTA at the three doses compared to control mice (OTA 0.21: *p* = 0.034 for unclassified *Bacteroidales* and for *Porphyromonadaceae*; OTA 0.5: *p* = 0.021 for unclassified *Bacteroidales* and for *Porphyromonadaceae*; OTA 1.5: *p* = 0.043 for unclassified *Bacteroidales* and *p* = 0.021 for *Porphyromonadaceae*; Figure 5b). *Streptococcaceae* and *Enterobacteriaceae* also showed a significant increase but only in the lower-dose OTA groups compared to control, while no changes occurred in the high-dose OTA group (OTA 0.21: *p* = 0.034 for *Streptococcaceae* and for *Enterobacteriaceae*; OTA 0.5: *p* = 0.043 for *Streptococcaceae* and *p* = 0.021 for *Enterobacteriaceae*; OTA 1.5: *p* > 0.05 for *Streptococcaceae* and *Enterobacteriaceae,* n.s.; Figure 5b). *Ruminococcaceae* decreased significantly in mice exposed to OTA at a dose of 0.5 mg/kg, although remained similar to control mice in mice exposed to OTA at 0.21 and 1.5 mg/kg (OTA 0.5: *p* = 0.029 different from control mice; Figure 5b). Finally, a significant decrease in the relative abundance of an unclassified *Cyanobacteria* was observed in mice exposed to the higher-dose OTA compared to control mice (*p* = 0.015; Figure 5b).

## 3. Discussion

In the present study, we aimed to investigate OTA gut microbiome toxicity in mice sub-chronically exposed for 28 days to OTA by daily oral gavage of different low doses of OTA. The study was carried out in mice as this species is considered to be the prime research model for microbiome studies [37,38]. The effect of other mycotoxins in gut microbiota has also been evaluated in mice [39,40].

The dose selection was made on the basis of the most recent mice studies reviewed by the European Food Safety Authority [27]. More concretely, the higher dose selected (1.5 mg/kg bw) was considered as the lowest observed adverse effect level (LOAEL) (in terms of kidney antioxidants response) of a 45-day oral study. In our experiments, in order to further mimic the low human exposure, two extra lower doses were also included (0.21 and 0.5 mg/kg bw).

The animals treated with 0.21, 0.5, or 1.5 mg/kg OTA did not show any clinical sign of toxicity (weekly observations) during the study, but the body weight of the 1.5 mg/kg OTA group showed a lower increase. Moreover, OTA was detectable in plasma samples in all the treated groups, showing a dose-dependent increase of OTA concentration [41].

We observed that exposure to low OTA doses induced substantial changes in the diversity and composition of gut microbiota. The unique study in mice used culture-dependent methods to investigate the effect of OTA on the growth of colonic probiotic bacteria [36]. This approach has great limitations since the majority of intestinal bacteria are widely considered to be unculturable and have never been isolated in the laboratory. The rapid rise of high-throughput sequencing methods, such as the one used in this study, provide an effective approach to study the composition of the host microbiota directly in their natural environments, avoiding the need for isolation and lab culture of individual species [42]. This study is the first to describe in depth the effects of oral exposure to different low doses of OTA on fecal microbiota in mice.

We found that OTA exposure caused marked changes in the gut microbial community structure, including the decrease in the diversity of fecal microbiota and the relative abundance of *Firmicutes*, as well as the increase in the relative abundance of *Bacteroidetes*. These results are in agreement with previous studies on other animal species [32,33]. Wang et al. observed these changes in ducks orally treated for 14 days with OTA (235 μg/kg) [32], while Zhai et al. fed ducklings with 2 mg/kg OTA-contaminated diet for 21 days [33]. However, changes in the relative abundance of *Bacteroidetes* and *Firmicutes* were not observed in the cecal microbiota of broilers intragastrically administrated with OTA 50 μg/kg for 21 days [34]. The inconsistence in the results could be associated with the different animal species used and their differences in the initial composition of the microbial communities, or could be due to the fact that the composition of gut microbiota is influenced by several factors including differences in OTA dose, genetic background [43], as well as the stress condition of the host [44]. In addition, microbiota has been described to vary dramatically along the length of the gut [45,46], which could also explain the differences observed. Only one study has investigated the effect of OTA in rodents [31]—in this study, F344 rats were orally treated with 70 and 210 μg/kg OTA for 28 days. In Guo’s study, the doses used did not showed strong signs of toxicity at the moment of the fecal collection (28 days) but were demonstrated to show the classical dose-dependent hallmarks of OTA toxicity after 13 weeks of treatment. As in our study, animals treated for 4 weeks with the high dose also showed a slightly lower increase of body weight. It should be noted when comparing both studies that, although the doses investigated are similar, mice are considered to be less sensitive to OTA toxicity than rats [27]. Fecal microbiota was analyzed at day 0 and 28 and relative changes over time were described. In agreement with our results, OTA treatment decreased alpha-diversity; however, there were important differences in gut microbiota composition compared with our data, which were likely associated with the animal species. Importantly, Guo et al. analyzed the microbiota composition at the family and genera level, and our study analyzed the bacteria composition at the phylum and family level. Their study highlighted the increase in *Lactobacillaceae* associated with OTA treatment, one of the most abundant families in rat gut microbiota [31]. In our samples *Lactobacillaceae* represented around 1% of total bacteria and we found a non-statistically significant increase in the groups treated with 0.5 and 1.5 mg/kg.

Interestingly, in our study, alterations on mouse fecal microbiota were observed in the lower-dose OTA groups, while high-dose OTA group microbiota was similar to that of the control group. Beta-diversity analysis also showed that lower-dose OTA groups were clearly separated from control and high-dose OTA groups. This phenomenon has been described previously with other mycotoxins, deoxynivalenol [39] and aflatoxin [40], in mice. Wang et al. observed a significant increase in the abundance of *Firmicutes* and particularly *Lactobacillus* genus in the low-dose group treated with deoxynivalenol, but the high-dose group remained similar to the control group [39]. In this study, as in our study, the high-dose group also showed a body weight decrease. Aflatoxin treatment increased the relative abundance of five genera, namely, *Peptostreptococcaceae*, *Allobaculum*, *Clostridium*, *Turicibacter*, and *Candidatus*, in the low- and medium-dose groups, but not in the high-dose group [40]. A potential explanation for the unusual dose–response effect of OTA upon microbiota could be associated with an antibiotic effect of OTA at low doses and a cytotoxic effect at higher doses. The antibiotic effect could affect the susceptible bacteria. OTA gut toxicity and immunotoxic effect may induce changes in mucus secretion, increased production of cytokines, and increased secretion of immunoglobulin A and antimicrobial peptides. All these changes caused by higher OTA levels may have a general impact in gut microbiota and could mask the antibacterial effect of OTA. However, the cause of the difference observed needs further investigation to elucidate the factor that led to this phenomenon.

We can predict that the observed decrease in the diversity and the changes in the community structure of the gut microbiota in OTA-exposed mice could be hazardous to the host’s health. Reductions in the diversity of the gut microbiota have been described in many studies on intestinal and immune diseases, such as Crohn’s disease [47] and ulcerative colitis [48]. In addition, the increase in *Bacteroidetes* together with the decrease in *Firmicutes* is one of the key changes in gut microbiota observed in elderly populations possibly related to aging [49,50].

An interesting feature of OTA-induced dysbiosis is the significant higher proportion of *Porphyromonadaceae* family within *Bacteroidetes* in the three OTA-treated groups. *Porphyromonadaceae* species appear to be less prevalent in healthy individuals [51] and have been exclusively detected in Crohn’s disease patients [47]. In addition, we found that OTA exposure also induced significant changes in other minor constituents of the gut microbiota in mice such as *Proteobacteria* species, which accounted for only 1.8% of total abundance. Specifically, our results showed a marked increase in the relative abundance of *Enterobactericeae*, a bacterial family within *Proteobacteria*, in OTA-treated mice. *Enterobactericeae* is a large family of Gram-negative bacteria that includes a number of pathogenic bacteria such as *Enterobacter* spp., *Salmonella* spp., and *Escherichia coli* that have been involved in intestinal inflammatory responses [52,53]. Therefore, the increased abundance of *Porphyromonadaceae* and *Enterobactericeae* could contribute to the intestinal inflammation, diarrhea, and other intestinal changes induced by OTA exposure [28,54].

Another hallmark of the dysbiosis induced by OTA is the decrease in the abundance of *Cyanobacteria* phylum in mice exposed to OTA at a dose of 1.5 mg/kg. Given that little is known about the functions and effects of *Cyanobacteria* on the gut bacterial community, it is difficult to speculate about the implications of the OTA-induced decrease of *Cyanobacteria* abundance.

The OTA-induced alterations of the gut microbiota composition also suggest diverse susceptibility of the different bacterial strains to OTA exposure. Thus, OTA could inhibit the growth of certain bacterial families that decreased following oral exposure to OTA. *Firmicutes* may be more susceptible than *Bacteroidetes* to the inhibitory effects of OTA. However, although *Firmicutes* are globally decreased in gut microbiota from OTA-treated mice, we found a family within the *Firmicutes* phylum, *Streptococcaceae*, which is known to exhibit high resistance to OTA. A previous culture-dependent study examined the susceptibility of different bacterial species to various mycotoxins, including OTA, and demonstrated that *Streptococcus agalactiae*, which belongs to the *Streptococcaceae* family, is resistant to the antibacterial effect of OTA [55].

On the other hand, there is a bidirectional interaction between mycotoxins and the intestinal microbiota in such a way that OTA induces changes in the intestinal microbiota, but in turn the microbes that reside in the gut participate in the process of elimination of mycotoxins through metabolism or binding to mycotoxins. It has been described that certain bacteria, mainly lactic acid bacteria (LAB), are able to detoxify OTA [56,57], and even preincubation of OTA with LAB reduces the toxic effects of the mycotoxin in human-derived liver cells [57]. Moreover, a previous in vitro study showed OTA transformation by animal microbiota [58]. Convincing evidence suggest that gut microbiota play a crucial role in OTA detoxification in rumiants [59]. The increased abundance of determined bacterial families in the fecal microbiota from OTA-exposed mice could be related to the degradation process of OTA to OTα [60], although further studies are required to prove this.

Finally, we cannot exclude the fact that OTA exposure could affect other bacteria not described in the present study since it is possible that some rare bacteria are not represented in the reference databases used or that they are at such a low level that are missed when applying filtering thresholds during data analysis. In addition, although the use of fecal samples for assessing the gut microbiome provides several advantages, they do not fully represent the microbes in the gut, particularly mucosal adherent microbes [61]. Moreover, as mentioned before, gut microbiota varies dramatically along the length of the gut [45,46]. Therefore, further studies involving sampling from different gut sections are required to gain a complete overview of the changes in gut microbiota induced by OTA exposure in mice.

Given the increasingly recognized role of the gut microbiota in human health coupled to its susceptibility to OTA exposure, our results would suggest that the toxic effects induced by OTA in organs different from intestine could be influenced by gut microbiome toxicity. In agreement with this, a recent study demonstrated that curcumin alleviated liver oxidative injury by modulating the alteration of gut microbiota induced by OTA in ducks [33]. Nevertheless, this hypothesis remains to be revealed and further studies are required to confirm it. More importantly, metabolic and functional studies would be helpful to improve our understanding of the OTA-induced effects on gut microbiota and gain new insights about possible mechanisms of OTA toxicity mediated by gut microbiota.

## 4. Conclusions

Our results demonstrated that sub-chronic exposure at low doses of OTA altered the structure and diversity of the gut microbial community in mice. Given the crucial role of gut microbiota in human health coupled with the capacity of OTA to induce gut microbiota toxicity, it is worth further investigating the mechanisms underlying OTA-induced gut microbiota alterations and the impact in OTA systemic toxic effects.

## 5. Materials and Methods

### 5.1. Animals

In compliance with the 3Rs for refining, reducing, and replacing animals for research purposes, we obtained the analyzed samples of the present study from a previous in vivo study [41], in which OTA levels in plasma and tissues and some general toxicity parameters (clinical examination and body weight) were evaluated. Eight-to nine-week-old male Balb/c mice (20–25 g) were purchased from Charles River (Wilmington, MA, USA). All animals were randomly distributed to the cages by a technician of the animal facilities, and before any procedure, the cages were selected randomly and randomized to each group by a person not involved in the study. Animals were housed in individual polycarbonate cages with stainless steel covers (4 mice per cage) with ad libitum access to standard pellet diet (Special Diet Service, UK; product code# 801010 RM1-A-P) and normal tap water, and were maintained in constant environmental conditions of humidity (55 ± 10%) and temperature (22 ± 2 °C) on a 12-h light/dark cycle. All the in vivo experiments as well as the investigators responsible for data collection and analysis were blinded. This study was approved by the Ethics Committee on Animal Experimentation of the Center of Biomedical Research of La Rioja (CIBIR, permit number LAE-02, date of approval 30 May 2016) and was conducted according to the National Institute of Health (NIH) Guide for the Care and Use of Laboratory.

### 5.2. OTA

OTA (1877, Lot 14181021-059M4100V) was purchased in powder from Sigma-Aldrich (Steinheim, Germany) and was dissolved in 0.10 M NaHCO_3_ (pH 7.4) for animal treatment. Then, OTA solutions were aliquoted and maintained at −20 °C until use.

### 5.3. Experimental Design and Sample Collection

Balb/c mice were acclimated for 1 week before the experiment, and randomly distributed into 4 dosing groups of 4 mice per group. Mice received repeated OTA administrations (0.21, 0.5, or 1.5 mg/kg) or vehicle (NaHCO_3_) daily for 28 days by oral gavage. The volume of the administration was 5 mL/kg, and therefore the animals were weighed daily in order to adjust the volume and the dose administrated to the animal weight. Mice were sacrificed by overdose of inhaled isoflurane at the end of the OTA treatment. Fecal samples were collected at sacrifice and were taken directly from the rectum. Mouse feces were put into sterile plastic tubes and processed immediately.

### 5.4. Bacterial Genomic DNA Extraction

Genomic DNA of gut microbiota was extracted from fecal samples collected from each individual mouse using the PowerFecal DNA Isolation kit (MO BIO Laboratories, Carlsbad, CA, USA, catalog no. 12830-50) following the manufacturer’s protocol. Briefly, immediately after collection, about 250 mg of feces was added to 2 mL dry bead tubes containing 750 μL of bead solution and gently vortexed. After C1 solution was added, the samples were briefly vortexed and incubated at 65 °C for 10 min. To aid in collision of the beads with microbial cells and optimize homogenization of the samples, we horizontally shook the bead tubes for 10 min and then subsequently centrifuged them at 10,000× *g* for 30 s. The supernatants were collected and transferred to the provided 2 mL collection tubes. The remainder of the protocol was followed as recommended by the manufacturer. All samples were eluted in 100 μL and stored at −20 °C until downstream application.

### 5.5. 16S rRNA Gene Sequencing

DNA samples extracted from fecal samples were analyzed in a Fragment Analyzer (Genomic DNA 50 Kb kit, AATI) to ensure their integrity, and quantified using a Qubit fluorometer (dsDNA HS Assay kit, Invitrogen, Carlsbad, CA, USA). From 12.5 ng of DNA of each sample, the library was prepared following the instructions of the 16S rRNA Metagenomic Sequencing Library Preparation (Illumina) protocol. Primer sequences cover the V3–V4 regions of the 16S rRNA gene. The following primers also include the Illumina adapters: 16S Amplicon PCR forward primer = 5′ (TCGTCGGCAGCGTCAGATGTGTATAAGAGACAGCCTACGGGNGGCWGCAG) and 16S Amplicon PCR reverse primer = 5′ (GTCTCGTGGGCTCGGAGATGTGTATAAGAGACAGGACTACHVGGGTATCTAATCC). The sequencing run was carried out by an Illumina sequencer (MiSeq, 2 × 300 bp, paired-end) (Illumina Inc., San Diego, CA, USA).

### 5.6. Analysis of Gut Microbiota Data 

The quality of the raw unprocessed reads was evaluated using the FastQC software (https://www.bioinformatics.babraham.ac.uk/projects/trim_galore/ Babraham Institute, 

Cambridge, UK). After removal of adapters by Trim Galore (https://www.bioinformatics.babraham.ac.uk/projects/trim_galore/), the quality of clean reads was re-evaluated with FastQC. Since the fragments sequenced for each of the samples were overlapped in their central region, the V3–V4 region of the 16S rRNA gene was partially reconstructed into fragments of approximately 550–580 bp. Reconstruction of full-length V3-V4 16S rRNA gene regions for taxonomic assignment and the determination of operational taxonomic units (OTUs) were carried out through the QIIME program (v1.9.1) [62] using the Greengenes database at 97% nucleotide identity (database version gg_13_5, https://greengenes.secondgenome.com/?prefix=downloads/greengenes_database/gg_13_5/).

Alpha-diversity indexes (Chao1, Shannon, and Simpson) were calculated using QIIME (v1.9.1), which generates multiple rarefactions on the OTU table at different sequencing depths, calculates the alpha diversity indexes at each depth, and finally generates rarefaction graphs for each index. Analysis was set out from the standardized and filtered table of OTUs to eliminate those OTUs that may be spurious and was carried out at OTU taxonomic levels. A threshold of 0.01% was applied, meaning that the OTU sequences with abundance below the 0.01% were assigned as spurious sequences and therefore removed from the analysis. The alpha-diversity indexes were statistically compared between groups through the Python script “compare_alpha_diversity.py” included in the QIIME v1.9.1 package. It performs a two-sample *t*-test by using a non-parametric (Monte Carlo) method and permutation value of 999. The *t*-test value and a *p*-value (Bonferroni correction) were obtained for each couple of defined groups.

Beta-diversity (Bray–Curtis dissimilarity) was calculated using QIIME v1.9.1. The OTU table normalization, applying the Cumulative Sum Scaling (CSS) method through the MetagenomeSeq package, was chosen as an alternative to the rarefaction one, in accordance with previous studies [63,64]. Principal coordinate analysis (PCoA) based on Bray–Curtis dissimilarity was developed and used to evaluate structure of mice fecal microbiota across the experimental groups. Results were plotted according to the first two principal components.

Finally, differential abundance analysis (comparisons among groups) was carried out by classical univariate analysis using Kruskal–Wallis or Mann–Whitney *U* tests. Statistical analysis was carried out using SPSS 19.0 (SPSS Inc., Chicago, IL, USA). *p*-value <0.05 was considered statistically significant.

## Figures and Tables

**Table 1:** Relative abundance of fecal microbiota in mice treated with different doses of OTA.

Phylum	Family	Control	OTA 0.21	OTA 0.5	OTA 1.5
Unclassified	Unclassified	0.47 ± 0.12	0.50 ± 0.05	0.46 ± 0.11	0.42 ± 0.05
*Bacteroidetes*	Unclassified *Bacteroidales*	0.32 ± 0.03	0.46 ± 0.09 *	0.44 ± 0.07 *	0.41 ± 0.07 *
	*Bacteroidaceae*	2.16 ± 0.30	3.45 ± 0.46	2.68 ± 0.66	2.37 ± 0.29
	*Porphyromonadaceae*	0.11 ± 0.01	0.20 ± 0.06 *	0.15 ± 0.02 *	0.19 ± 0.02 *
	*Prevotellaceae*	1.14 ± 0.06	1.19 ± 0.04	1.22 ± 0.07	1.17 ± 0.04
	*Rikenellaceae*	4.75 ± 0.10	5.09 ± 0.23	4.72 ± 0.33	4.77 ± 0.36
	*S24-7*	24.87 ± 1.42	28.62 ± 2.29	28.71 ± 2.41	25.60 ± 1.70
	*Odoribacteraceae*	0.63 ± 0.32	0.78 ± 0.03	0.82 ± 0.05	0.77 ± 0.16
*Cyanobacteria*	Unclassified *Cyanobacteria*	0.90 ± 0.08	0.94 ± 0.04	0.84 ± 0.06	0.68 ± 0.07 *
*Deferribacteres*	*Deferribacteraceae*	0.47 ± 0.09	0.45 ± 0.10	0.48 ± 0.24	0.47 ± 0.04
*Firmicutes*	*Lactobacillaceae*	0.92 ± 0.30	0.99 ± 0.76	1.52 ± 0.13	1.44 ± 0.08
	*Streptococcaceae*	0.02 ± 0.03	0.24 ± 0.12 *	0.19 ± 0.18 *	0.03 ± 0.03
	Unclassified *Clostridiales*	34.55 ± 1.47	28.60 ± 2.44	30.35 ± 2.30	32.71 ± 2.79
	*Clostridiaceae*	0.28 ± 0.12	0.40 ± 0.09	0.36 ± 0.10	0.28 ± 0.04
	*Dehalobacteriaceae*	0.57 ± 0.03	0.62 ± 0.05	0.57 ± 0.05	0.54 ± 0.06
	*Lachnospiraceae*	8.05 ± 0.96	7.58 ± 0.45	8.63 ± 1.69	9.13 ± 1.29
	*Peptococcaceae*	0.17 ± 0.09	0.20 ± 0.04	0.20 ± 0.14	0.17 ± 0.05
	*Ruminococcaceae*	15.61 ± 0.66	15.58 ± 0.48	13.40 ± 1.43 *	14.96 ± 0.82
	*Mogibacteriaceae*	0.14 ± 0.09	0.19 ± 0.03	0.15 ± 0.04	0.17 ± 0.03
	*Erysipelotrichaceae*	0.62 ± 0.22	0.32 ± 0.07	0.59 ± 0.17	0.56 ± 0.10
*Proteobacteria*	Unclassified *Alphaproteobacteria*	0.81 ± 0.20	0.84 ± 0.21	0.73 ± 0.15	0.68 ± 0.27
	*Alcaligenaceae*	0.44 ± 0.04	0.41 ± 0.02	0.44 ± 0.04	0.38 ± 0.03
	*Desulfovibrionaceae*	0.39 ± 0.06	0.40 ± 0.09	0.42 ± 0.02	0.43 ± 0.06
	*Enterobacteriaceae*	0.08 ± 0.02	0.36 ± 0.15 *	0.36 ± 0.31 *	0.09 ± 0.07
*TM7*	*F16*	0.23 ± 0.07	0.21 ± 0.01	0.27 ± 0.01	0.23 ± 0.02
*Tenericutes*	*Anaeroplasmataceae*	0.73 ± 0.07	0.79 ± 0.28	0.76 ± 0.18	0.87 ± 0.06
	Unclassified *Mollicutes*	0.54 ± 0.30	0.59 ± 0.15	0.54 ± 0.05	0.48 ± 0.07

Data are shown as mean ± standard deviation (*n* = 3–4). Statistical significance was determined by non-parametric Kruskal–Wallis test followed by Mann–Whitney *U* test; * *p* <0.05 significantly different from control mice.

## Data Availability

All primary data and materials will be made available under request.
